# Oral Pathogen *Fusobacterium nucleatum* Coaggregates With *Pseudomonas aeruginosa* to Modulate the Inflammatory Cytotoxicity of Pulmonary Epithelial Cells

**DOI:** 10.3389/fcimb.2021.643913

**Published:** 2021-03-19

**Authors:** Qian Li, Hongyan Wang, Lisi Tan, Shuwei Zhang, Li Lin, Xiaolin Tang, Yaping Pan

**Affiliations:** ^1^ Liaoning Provincial Key Laboratory of Oral Diseases, Department of Oral Biology, School and Hospital of Stomatology, China Medical University, Shenyang, China; ^2^ Liaoning Provincial Key Laboratory of Oral Diseases, Department of Periodontics, School and Hospital of Stomatology, China Medical University, Shenyang, China

**Keywords:** *Pseudomonas aeruginosa*, *Fusobacterium nucleatum*, chronic obstructive pulmonary disease, bacterial coaggregation, pulmonary epithelial cells, inflammatory cytotoxicity

## Abstract

Chronic obstructive pulmonary disease (COPD) is the third leading cause of mortality worldwide, and inflammatory damage induced by bacterial infections is an important contributor to the etiology of COPD. *Fusobacterium nucleatum*, a recognized periodontal pathogen, is considered as a biomarker of lung function deterioration of COPD patients coinfected with *Pseudomonas aerugionsa*, but the underlying mechanism is still unclear. This study established single- and dual-species infection models, bacterial simultaneous and sequential infection models, and found that *F. nucleatum* could coaggregate with *P. aeruginosa* to synergistically invade into pulmonary epithelial cells and transiently resist *P. aeruginosa*-induced cytotoxic damage to amplify IL-6 and TNF-α associated inflammation in pulmonary epithelial cells simultaneously infected with *P. aeruginosa* and *F. nucleatum*. Furthermore, *F. nucleatum* pretreatment or subsequential infection could maintain or even aggravate *P. aeruginosa*-induced inflammatory cytotoxicity of pulmonary epithelial cells. These results indicate that oral pathogen *F. nucleatum* coaggregates with *P. aeruginosa* to facilitate bacterial invasion and modulates the inflammatory cytotoxicity of pulmonary epithelial cells, which may contribute to lung function deterioration of COPD patients accompanied with *P. aeruginosa* and *F. nucleatum* coinfection.

## Introduction

Chronic obstructive pulmonary disease (COPD) is the third leading cause of mortality worldwide, characterized by persistent airflow limitation and respiratory symptoms including dyspnea, cough and/or sputum production (Singh et al., 2019). 54.7% of patients with COPD exacerbations admitted to the hospital are associated with respiratory bacterial infection, (Papi et al., 2006). *Pseudomonas aeruginosa*, a ubiquitous opportunistic pathogen, is one of the most prevalent bacteria responsible for respiratory infection in COPD patients (Choi et al., 2019). *P. aeruginosa* is associated with prolonged hospitalization, increased exacerbation rate and poor lone-term prognosis in COPD patients (Garcia-Vidal et al., 2009; Almagro et al., 2012; Eklof et al., 2020).

In 1999, Dr. Scannapieco first proposed that oral microbiota is closely related to respiratory infections (Scannapieco, 1999). The dynamic and polymicrobial dental plaque biofilm has been proved as a reservoir of respiratory pathogens, direct inhalation of the shedding of dental plaque colonized by respiratory pathogens into lung is one of the most possible mechanisms involved in the association between oral microbiota and respiratory diseases (Tan et al., 2014; Sands et al., 2017). *Fusobacterium nucleatum*, an oral commensal and periodontal pathogen, is ubiquitous in the oral cavity of healthy and diseased individuals. *F. nucleatum* acts as a coaggregation bridge between early and late colonizing bacteria in the polymicrobial dental plaque biofilm, and affects the composition and architecture of supra- and subgingival biofilm (Thurnheer et al., 2019). Recent studies demonstrate that *F. nucleatum* colonizes in the respiratory tract, not only leads to respiratory infection in patients with chronic diseases such as tumors and diabetes (Yang et al., 2011; Bailhache et al., 2013; Shamriz et al., 2015), but also causes endobronchial lesion in healthy children (Gedik et al., 2014). Meanwhile, our previous study finds that *F. nucleatum* and *P. aeruginosa* frequently coexisted in the respiratory tract of patients with COPD exacerbation, and as the number of *F. nucleatum* in the respiratory tract microbiota increases, their lung function declines (Li et al., 2020). These results suggest that *F. nucleatum* and *P. aeruginosa* may play a synergistic role in respiratory infection of COPD patients, but the mechanism underlying this is still unclear.

The ability to adhere and invade host cells is a virulence property of certain bacteria. Although *P. aeruginosa* is usually considered an extracellular pathogen, some studies have demonstrated that *P. aeruginosa* can invade a variety of epithelial cells *via* an endocytic process dependent on actin microfilaments (Fleiszig et al., 1994; Fleiszig et al., 1995). The invasive *P. aeruginosa* induces membrane blebs in epithelial cells, which are utilized as a niche for bacterial intracellular replication and motility (Angus et al., 2008). In contrast, *F. nucleatum* adheres to and invades epithelial cells *via* a zipping mechanism relying on its outer membrane proteins to bind to the cell-surface receptors (Han et al., 2000). At the same time, *F. nucleatum* can coaggregate with almost all of the oral bacteria dependent on its outer membrane proteins (Kaplan et al., 2009; Loozen et al., 2014; Coppenhagen-Glazer et al., 2015). *F. nucleatum* not only enhances the adhesion and invasion of invasive bacteria such as *Porphyromonas gingivalis* and *Aggregatibacter actinomycetemcomitans* to tissue cells (Li et al., 2015), but also transports noninvasive bacteria such as *Streptococcus cristatus* and *Streptococcus sanguis* into epithelial cells (Edwards et al., 2006). It is still unclear whether *F. nucleatum* will affect the infection of *P. aeruginosa* to pulmonary epithelial cells due to interspecies coaggregation in COPD patients accompanied with coinfection of *F. nucleatum* and *P. aeruginosa*.

Mucosal epithelium is not only the first physical barrier between host and bacteria, but also an active participant in natural immunity and inflammation (Proud and Leigh, 2011). IL-1β is considered a biomarker of bacteria-associated exacerbation of COPD, and TNF-α and IL-6 are the potential markers of IL-1β-associated exacerbation of COPD (Bafadhel et al., 2011; Damera et al., 2016). Vos et al. found that *P. aeruginosa* infection of human bronchial epithelial cells can significantly induce the expression of IL-1 family members IL-1β and IL-1F9 (Vos et al., 2005). *P. aeruginosa*-derived flagellin induces IL-6 and IL-8 production in bronchial epithelial cells through the phosphorylation of p38, ERK and JNK, which partially explains the underlying mechanism of *P. aeruginosa* causing acute exacerbation of COPD (Nakamoto et al., 2019). Hayata et al. report that *F. nucleatum* induces the productions of proinflammatory cytokines IL-6 and IL-8 by the bronchial and pharyngeal epithelial cells, which may trigger exacerbation of COPD (Hayata et al., 2019). Therefore, we speculate that coinfection of *P. aeruginosa* and *F. nucleatum* may induce cytokine release dysregulation in pulmonary epithelial cells, we plan to establish bacterial simultaneous and sequential infection models to simulate three types of clinical mixed-infection modes in order to fully confirm our scientific hypothesis, and to further reveal the possible mechanism of *P. aeruginosa* and *F. nucleatum* coinfection causing lung function decline in COPD patients.

On the basis of the above thinking and hypothesis, the aim of this study was to explore the potential mechanism by which *F. nucleatum* cooperated with *P. aeruginosa* to exacerbate COPD. First, we investigated the effect of bacterial coinfection on bacterial adhesion and invasion ability, and the role of bacterial coaggregation in this process. Second, we established bacterial simultaneous and sequential infection models to simulate three types of clinical mixed-infection modes, and detected the effect of the combined infection with *P. aeruginosa* and *F. nucleatum* on inflammation and damage of pulmonary epithelial cells.

## Materials and Methods

### Bacteria Culture

*F. nucleatum* (ATCC 25586) and *P. aeruginosa* PAO1 strain (ATCC BAA-47) were obtained from American Type Culture Collection (ATCC). *F. nucleatum* was grown in tryptic soy broth (TSB. Becton, Dickinson and Company, Sparks, MD, USA) supplemented with 5% defibrinated sheep blood, 10mg/ml hemin and 5mg/ml menadione in anaerobic atmosphere (10% H_2_, 10% CO_2_, 80% N_2_) at 37 °C (Yu et al., 2017; Wu et al., 2018). *P. aeruginosa* was cultured in TSB under aerobic conditions at 37°C.

### Bacterial Aggregation Assay

*P. aeruginosa* and *F. nucleatum* were standardized in sterile coaggregation buffer (150 mM NaCl, 1 mM Tris, 0.1 mM CaCl_2_ and 0.1 mM MgCl_2_) to give a final cell density of 1×10^9^ colony-forming units per mL (CFUs/mL). Equal numbers of single- or dual-species bacterial cells were suspended and vortexed for 30 s in a reaction tube (T=0 h). The tubes were incubated at room temperature for 1 h to allow aggregation (T=1 h). A visual rating scale of 0-4 was used to grade the reaction. “0” indicates an evenly turbid suspension with no visible aggregates, meaning no coaggregation; “1+” indicates turbid supernatant with finely dispersed coaggregates; “2+” indicates definite coaggregates that do not precipitate immediately; “3+” indicates slightly turbid supernatant with formation of large precipitating coaggregates; and “4+” indicates complete sedimentation with a clear supernatant (Cisar et al., 1979; Shimazu et al., 2016). In addition, a spectrophotometric assay was employed to determine the percentage of bacterial auto- and coaggregation (Cisar et al., 1979; Kaplan et al., 2009). The optical density of bacterial suspensions at 600 nm wavelength (OD600) were measured at the time points of 0 and 1 h. Percentage aggregation was calculated using the following equation: % autoaggregation or coaggregation =(OD600_(T0)_-OD600_(T1)_)/OD600_(T0)_ ×100. Percentage aggregation were classified as high (more than 40%), intermediate (30-40%) and low aggregation (less than 30%).

#### Phase Contrast Microscopy

Auto- and coaggregation of the test bacterial species were also studied by phase contrast microscopy. After preparing bacterial suspensions with or without partner species in coaggregation buffer for 1 h as described above, 10 μL from each preparation was mounted on a microscopic glass slide with a coverslip and photographed by phase contrast microscope (Olympus, Japan).

### Cell Culture

Human pulmonary epithelial cell line A549 was obtained from ATCC and cultured in Dulbecco’s modified Eagle’s medium (DMEM. HyClone Laboratories, Logan, UT, USA) supplemented with 10% fetal bovine serum (FBS. HyClone Laboratories, Logan, UT, USA), 100 U/mL penicillin and 100 mg/mL streptomycin in a humidified 37°C incubator with 5% CO_2_.

### Bacterial Infection of Pulmonary Epithelial Cells

The *in vitro* bacterial infection model used in this study was performed as described previously (Pan et al., 2009; Li et al., 2019). Briefly, A549 cells were seeded into 24-well tissue culture plates at a density of 10^5^ cells/well. Cells were inoculated and grown for 24 h to allow the cells to reach confluence. Logarithmic growth phase bacteria were harvested and resuspended to 10^7^ CFUs/mL in DMEM without antibiotics. A549 cells were incubated with *F. nucleatum* and *P. aeruginosa* alone or together at multiplicity of infection (MOI, bacteria: epithelial cells) of 10, 50 or 100 in DMEM without antibiotics. For bacterial simultaneous infection model, A549 cells were incubated with both *F. nucleatum* (MOI 100) and *P. aeruginosa* (MOI 100) at the same times in DMEM without antibiotics. For bacterial sequential infection model, A549 cells were treated with *F. nucleatum* (MOI 100) or *P. aeruginosa* (MOI 100) in DMEM without antibiotics for 12 h, and then added the other bacteria (MOI 100) to continue infection for 12 h. In order to exclude the effect of the increased bacterial load, the single *P. aeruginosa* or *F. nucleatum* infection groups at a MOI of 200 were used as controls in the bacterial simultaneous infection assay, and the sequential inoculation of the same bacteria were used as controls in the bacterial sequential infection assay.

### Antibiotic Protection Adhesion and Invasion Assay

Assays of bacterial adherence and invasion were performed using methods previously described (Pan et al., 2009). Briefly, bacteria were harvested, washed, and resuspended in DMEM without antibiotics. A549 cells were infected with bacteria in DMEM without antibiotics in the humidified 37°C incubator with 5% CO_2_ for 3 h, and washed with phosphate-buffered saline (PBS). For determining total adhesion and invasion levels (attachment levels), cells were lysed with sterile distilled water for 30 min, then dilutions of the lysate were plated and cultured anaerobically on TSB agar supplemented with 5% defibrinated sheep blood, 10mg/ml hemin and 5mg/ml menadione to determine CFUs for *F. nucleatum*, or cultured aerobically on TSB agar to determine CFUs for *P. aeruginosa*. For invasion assay, extracellular bacteria were killed with 200 μg/mL gentamicin (Sigma, St. Louis, MO, USA) and 300 μg/mL metronidazole (Sigma, St. Louis, MO, USA) for 1 h. Cellular lysate were diluted and cultured to determine CFUs. The number of bacterial attachment or invasion is equal to CFUs divided by the number of cells.

### Scanning Electron Microscopy (SEM)

A549 cells were infected with *P. aeruginosa* (MOI 100) and *F. nucleatum* (MOI 100) alone or together in DMEM without antibiotics for 3 h, washed with PBS, and fixed with 2.5% glutaraldehyde (BioChemika, Fluka, Switzerland). A549 cells were gradually dehydrated with ethanol and smeared onto copper plates followed by gold sputtering, and images were acquired using SEM (Inspect F50, FEI Company, USA) to observe the bacterial adhesion and cell morphology.

### CCK-8 Cell Viability Assay

Cell counting kit (Dojindo Molecular Technologies, Inc., Kumamoto, Japan) was used to assess cell proliferation according to the manufacturer’s manual. Briefly, A549 cells were infected with *P. aeruginosa* and *F. nucleatum* alone (MOI 10, 50 or 100) or together (MOI 100) in DMEM without antibiotics for 4, 8, 16 and 24 h. 10 μL of CCK-8 solution was added to 100 μL of medium solution and incubated for 1 h at 37°C. OD450 was measured with a microplate reader (Infinite M200, Tecan, Austria) to reflect the cell proliferation.

### Cytotoxicity Assessment by Lactate Dehydrogenase (LDH) Leakage Assay

LDH activity was monitored by the Pierce™ LDH cytotoxicity assay kit (Thermo Scientific, USA) in accordance with the manufacturer’s recommendations. A549 cells were exposed to *P. aeruginosa* and *F. nucleatum* alone (MOI 10, 50 or 100) or together (MOI 100) in DMEM without antibiotics for the desired time, the culture supernatant was harvested and centrifuged at 12000 g for 5 min, and 50 μL supernatant was mixed with 50 μL Reaction mixture and incubated at room temperature in the dark for 30 min. 50 μL stop solution was added to stop reaction. OD490 and OD680 were measured with a microplate reader (Infinite M200, Tecan, Austria). LDH activity = OD490-OD680.

### Live/Dead Assay by Calcein Acetoxymethyl Ester/Propidium Iodide (Calcein-AM/PI) Staining

The effects of *P. aeruginosa* and *F. nucleatum* on the viability or cytotoxicity of pulmonary epithelial cells were evaluated using the Calcein-AM/PI Double Stain Kit (Molecular Probes, Eugene, OR, USA). In brief, A549 cells were exposed to *P. aeruginosa* and *F. nucleatum* alone or together in DMEM without antibiotics for the desired time, washed with PBS. Cells were stained with 200 μL Calcein-AM/PI stain working solution at 37°C for 15 min. Living cells (green cytoplasmic fluorescence) and dead cells (red nucleus fluorescence) were immediately observed by fluorescence microscope.

### Enzyme-Linked Immunosorbent Assay (ELISA)

A549 cells were exposed to *P. aeruginosa* and *F. nucleatum* alone or together in DMEM without antibiotics for the desired time, the culture supernatant was harvested and centrifuged at 12000 g for 5 min. IL-1β, IL-6 and TNF-α levels in the culture supernatant were determined *via* ELISA kits (Invitrogen, Cambrillo, USA) according to the manufacturer’s protocol. After the procedure, plates were read on the spectrometer at 450 nm wavelength. The results were converted to numeric values using standard curves.

### Statistical Analysis

All experiments were performed in triplicate and repeated three times. All values were presented as mean ± standard deviation (SD) and analyzed by analysis of variance followed by multiple comparisons test using GraphPad Prism version 7.00 (GraphPad Software, La Jolla, CA, USA). Differences were considered statistically significant at *P*<0.05.

## Results

### The Interaction Between *F. nucleatum* and *P. aeruginosa*: *F. nucleatum* Coaggregates With *P. aeruginosa* and Adheres to Pulmonary Epithelial Cells

**Figure 1:**
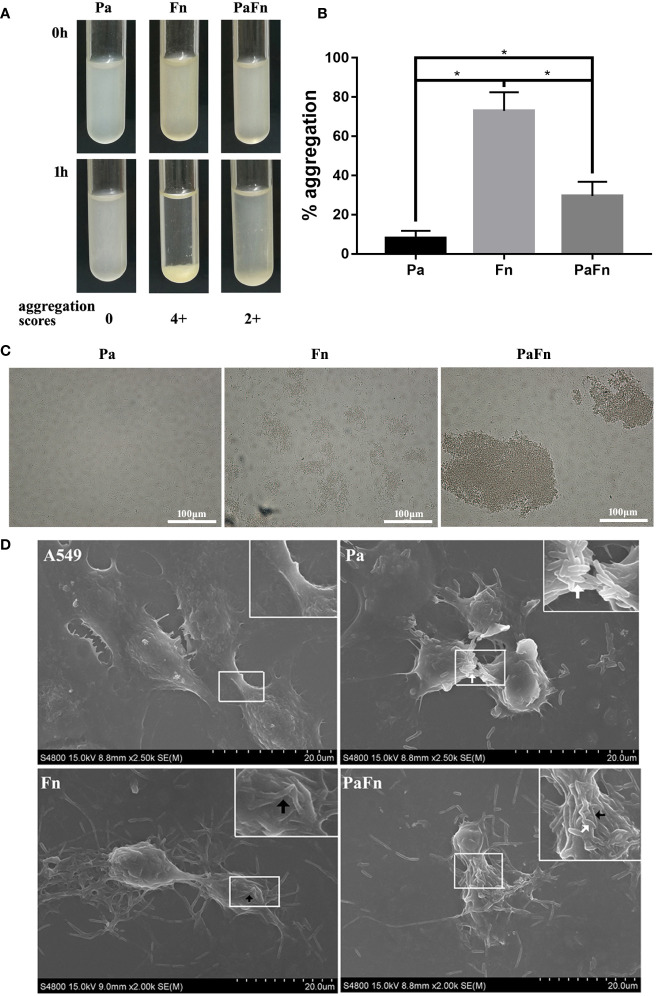
*F. nucleatum* coaggregates with *P. aeruginosa* and adheres to pulmonary epithelial cells. **(A)** Photographs of autoaggregation and coaggregation of *P. aeruginosa* and *F. nucleatum*. The aggregation score of each bacterial group was indicated below the photograph. **(B)** Percentage aggregation of *P. aeruginosa* and *F. nucleatum*. *P < 0.05, statistics were achieved by analysis of variance followed by Tukey’s multiple comparisons test. **(C)** Representative images of auto- and coaggregation of *P. aeruginosa* and *F. nucleatum* observed by phase contrast microscope. **(D)** Representative SEM images of pulmonary epithelial cells infected with *P. aeruginosa* and *F. nucleatum* alone or together. A549 cells were infected with *P. aeruginosa* (MOI 100) and *F. nucleatum* (MOI 100) alone or together for 3 h. White arrow indicates *P. aeruginosa*, and black arrow indicates *F. nucleatum*. Pa, *P. aeruginosa*; Fn, *F. nucleatum*.

To detect the relationship between *P. aeruginosa* and *F. nucleatum*, bacterial aggregation assay was performed. The profiles of microbial aggregation of *P. aeruginosa* and *F. nucleatum* were showed in **Figures 1A–C**. *P. aeruginosa* displayed a poor autoaggregation with an autoaggregation score of “0” and a low percentage autoaggregation (8.1 ± 3.66%) (**Figures 1A–C**). *F. nucleatum* showed a strong autoaggregation with an autoaggregation score of “4+” and a high percentage autoaggregation (72.99 ± 9.43%) (**Figures 1A–C**). Moreover, *F. nucleatum* and *P. aeruginosa* were intermediate coaggregators with an autoaggregation score of “2+” and a percentage autoaggregation of 30.52 ± 7.28% (**Figures 1A–C**).

To explore whether bacterial coaggregation affect the form of bacterial adherence to host cells, bacterial adhesion assay and SEM were performed. **Figure 1D** demonstrated that *P. aeruginosa* mainly attached to the cellular junction of pulmonary epithelial cells, which shrunk and became round (**Figure 1D**). *F. nucleatum* auto-aggregated to form a bacterial network and attached to the cellular surface of pulmonary epithelial cells, which remained stretched (**Figure 1D**). When *P. aeruginosa* and *F. nucleatum* coinfected pulmonary epithelial cells, *F. nucleatum* coaggregated with *P. aeruginosa* and adhered to the cellular junction and surface of pulmonary epithelial cells, which shrunk and became round (**Figure 1D**).

### The Model of Pulmonary Epithelial Cells Simultaneously Infected With *P. aeruginosa* and *F. nucleatum*

#### Coinfection with *P. aeruginosa* and *F. nucleatum* Promotes Each Bacterial Invasion of Pulmonary Epithelial Cells

**Figure 2:**
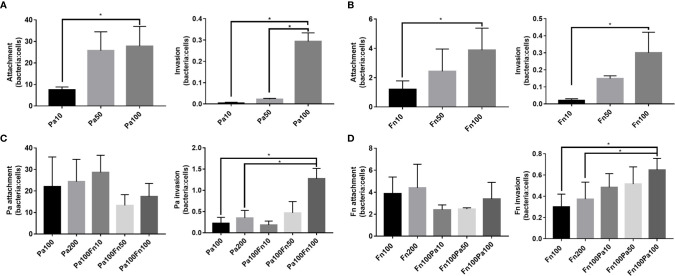
The adherence and invasion of *P. aeruginosa* and *F. nucleatum* to pulmonary epithelial cells. **(A)** The attachment and invasion of *P. aeruginosa* to pulmonary epithelial cells. A549 cells were infected with different MOI (10, 50, 100) of *P. aeruginosa*. **(B)** The attachment and invasion of *F. nucleatum* to pulmonary epithelial cells. A549 cells were infected with different MOI (10, 50, 100) of *F. nucleatum*. **(C)** The effect of *F. nucleatum* on the attachment and invasion of *P. aeruginosa*. A549 cells were simultaneously infected with *P. aeruginosa* (MOI 100) and *F. nucleatum* (MOI 10, 50, 100), and the single *P. aeruginosa* infection groups (MOI 100, 200) were used as control. **(D)** The effect of *P. aeruginosa* on the attachment and invasion of *F. nucleatum*. A549 cells were simultaneously infected with *F. nucleatum* (MOI 100) and *P. aeruginosa* (MOI 10, 50, 100), and the single *F. nucleatum* infection groups (MOI 100, 200) were used as control. *P < 0.05, statistics were achieved by analysis of variance followed by Sidak’s multiple comparisons test. Pa, *P. aeruginosa*; Fn, *F. nucleatum*.

To explore the effect of *P. aeruginosa* and *F. nucleatum* coinfection on bacterial attachment and invasion, pulmonary epithelial cells were infected with *P. aeruginosa* and/or *F. nucleatum* at different MOI (10, 50, 100). **Figures 2A, B** demonstrated that both *P. aeruginosa* and *F. nucleatum* could adhere to and invade the pulmonary epithelial cells, and as the bacterial MOI value increased, the number of *P. aeruginosa* and *F. nucleatum* attaching to and invading pulmonary epithelial cells increased. When the pulmonary epithelial cells were simultaneously infected with *P. aeruginosa* and *F. nucleatum* at different MOI (10, 50, 100), *F. nucleatum* did not affect the attachment number of *P. aeruginosa* to pulmonary epithelial cells, but *F. nucleatum* (MOI=100) significantly enhanced the invasion number of *P. aeruginosa* into pulmonary epithelial cells (**Figure 2C**). Meanwhile, *P. aeruginosa* did not affect the attachment number of *F. nucleatum* to pulmonary epithelial cells, but *P. aeruginosa* (MOI=100) significantly enhanced the invasion number of *F. nucleatum* into pulmonary epithelial cells (**Figure 2D**). In order to prove that interaction between these two bacterial species promotes each bacterial invasion of pulmonary epithelial cells, the single *P. aeruginosa* or *F. nucleatum* infection groups at a MOI of 200 were used as controls. **Figures 2C, D** demonstrated that there was not significantly difference in the number of bacterial attachment and invasion between Pa100 and Pa200 groups, and between Fn100 and Fn200 groups.

#### *F. nucleatum* Coinfection Transiently Resists *P. aeruginosa*-Induced Cytotoxicity of Pulmonary Epithelial Cells

**Figure 3:**
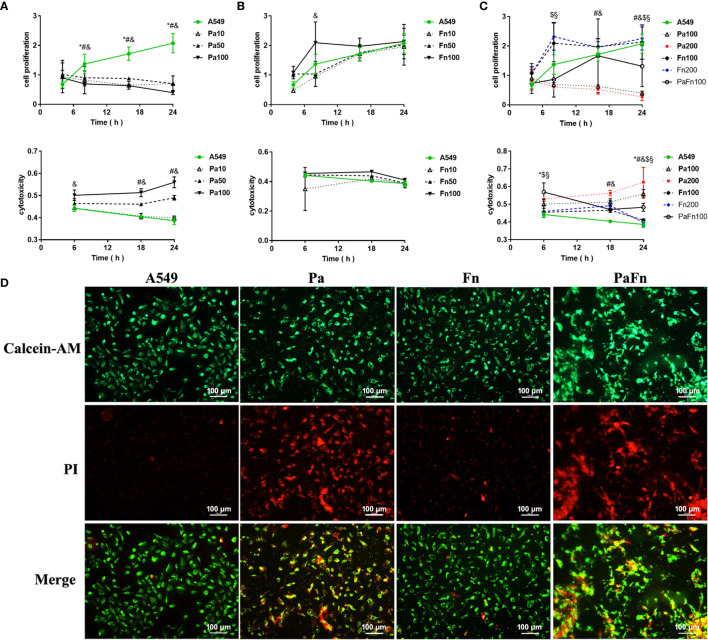
The effect of *P. aeruginosa* and *F. nucleatum* on cellular proliferation and cytotoxicity. **(A)** The effect of *P. aeruginosa* on cell proliferation and cytotoxicity of pulmonary epithelial cells. A549 cells were infected with different MOI (10, 50, 100) of *P. aeruginosa*. * Pa10 compared with A549, # Pa50 compared with A549, & Pa100 compared with A549. **(B)** The effect of *F. nucleatum* on cell proliferation and cytotoxicity of pulmonary epithelial cells. A549 cells were infected with different MOI (10, 50, 100) of *F. nucleatum*. * Fn10 compared with A549, # Fn50 compared with A549, & Fn100 compared with A549. **(C)** The effect of the simultaneous infection of *P. aeruginosa* and *F. nucleatum* on cell proliferation and cytotoxicity of pulmonary epithelial cells. A549 cells were simultaneously infected with *P. aeruginosa* (MOI 100) and *F*. *nucleatum* (MOI 100), and the single *P. aeruginosa* or *F. nucleatum* infection groups (MOI 100, 200) were used as control. * A549 compared with PaFn100, # Pa100 compared with PaFn100, & Pa200 compared with PaFn100, $ Fn100 compared with PaFn100, § Fn200 compared with PaFn100. */#/&/$/§ *P*<0.05, statistics were achieved by analysis of variance followed by Dunnett’s multiple comparisons test. **(D)** Representative fluorescence images of pulmonary epithelial cells infected with *P. aeruginosa* and *F. nucleatum* alone or together. A549 cells were infected with *P. aeruginosa* (MOI 100) and *F. nucleatum* (MOI 100) alone or together for 24 h. Pa, *P. aeruginosa*; Fn, *F. nucleatum*.

To exploit the effect of *P. aeruginosa* and *F. nucleatum* coinfection on the biological activity of pulmonary epithelial cells, cell proliferation and cytotoxicity were examined by CCK8 assay and LDH activity assay, respectively. Compared with the control (A549), *P. aeruginosa* (MOI 10, 50, 100) significantly inhibited the proliferation of pulmonary epithelial cells (**Figure 3A**). When the MOI of *P. aeruginosa* were 50 and 100, *P. aeruginosa* significantly induced cytotoxicity of pulmonary epithelial cells, and the cytotoxicity increased with the extension of infection time (**Figure 3A**). In contrast, *F. nucleatum* had no effect on the cytotoxicity of pulmonary epithelial cells, but enhanced the proliferation of pulmonary epithelial cells, which were infected by *F. nucleatum* at MOI of 100 at 8 h (**Figure 3B**). However, when pulmonary epithelial cells were simultaneously infected with *P. aeruginosa* and *F. nucleatum*, cell proliferation activity was lower than the single *F. nucleatum*-infected groups but higher than the single *P. aeruginosa*-infected groups after 12 h (**Figure 3C**). Meanwhile, the combined infection of *P. aeruginosa* and *F. nucleatum* significantly enhanced the cytotoxic response compared to A549 and single *F. nucleatum*-infected groups, but the cytotoxicity level of the combined infection group was lower than that of the single *P. aeruginosa* groups after 12 h (**Figure 3C**). In order to eliminate the effect of the increased bacterial load on cell proliferation and cytotoxicity, the single *P. aeruginosa* or *F. nucleatum* infection groups at a MOI of 200 were used as controls. **Figure 3C** demonstrated there was not significantly difference in cell proliferation and cytotoxicity of pulmonary epithelial cells between Pa100 and Pa200 groups, and between Fn100 and Fn200 groups.

In order to observe the cell viability or cytotoxicity intuitively, pulmonary epithelial cells were labeled with Calcein-AM and PI dyes. **Figure 3D** demonstrated that pulmonary epithelial cells infected with *F. nucleatum* were mainly live, but the *P. aeruginosa* alone or combination with *F. nucleatum* groups were mainly dead cells at 24 h.

#### *P. aeruginosa* and *F. nucleatum* Coinfection Enhances IL-6 and TNF-α Expression in Pulmonary Epithelial Cells Compared With Single *P. aeruginosa* Infection

**Figure 4:**
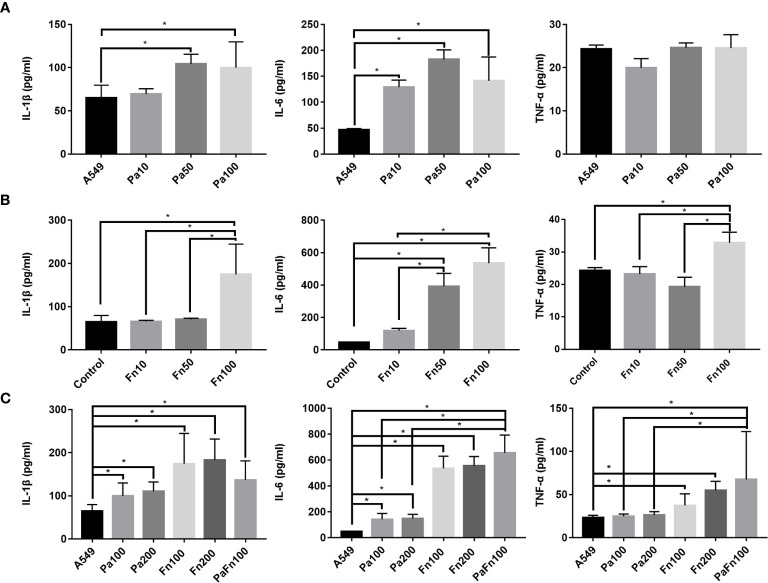
The effect of *P. aeruginosa* and *F. nucleatum* on IL-1β, IL-6 and TNF-α productions. **(A)** The effect of *P. aeruginosa* on IL-1β, IL-6 and TNF-α productions of pulmonary epithelial cells. A549 cells were infected with different MOI (10, 50, 100) of *P. aeruginosa* for 24 h. **(B)** The effect of *F. nucleatum* on IL-1β, IL-6 and TNF-α productions of pulmonary epithelial cells. A549 cells were infected with different MOI (10, 50, 100) of *F. nucleatum* for 24 h. **(C)** The effect of the simultaneous infection of *P. aeruginosa* and *F. nucleatum* on IL-1β, IL-6 and TNF-α productions of pulmonary epithelial cells. A549 cells were simultaneously infected with *P. aeruginosa* (MOI 100) and *F. nucleatum* (MOI 100) for 24 h, and the single *P. aeruginosa* or *F. nucleatum* infection groups (MOI 100, 200) were used as control. *P < 0.05, statistics were achieved by analysis of variance followed by Sidak’s multiple comparisons test. Pa, *P. aeruginosa*; Fn, *F. nucleatum*.

To detect the effect of *P. aeruginosa* and *F. nucleatum* coinfection on inflammatory response of pulmonary epithelial cells, the expressions of IL-1β, IL-6 and TNF-α were examined by ELISA. Compared with the control group (A549), *P. aeruginosa* significantly increased the secretion of IL-1β and IL-6 in pulmonary epithelial cells, but there was not significant difference in IL-1β and IL-6 secretion levels between different MOI groups (**Figure 4A**). *P. aeruginosa* had no significant effect on TNF-α secretion of pulmonary epithelial cells (**Figure 4A**). **Figure 4B** showed that compared with the control group (A549), *F. nucleatum* (MOI 100) significantly enhanced the secretion of IL-1β, IL-6 and TNF-α, and the secretion levels of IL-1β, IL-6 and TNF-α in *F. nucleatum* MOI 100 group were higher than those of *F. nucleatum* MOI 10 and/or MOI 50 groups. Moreover, *P. aeruginosa* and *F. nucleatum* coinfection significantly enhanced the secretion of IL-1β, IL-6 and TNF-α in pulmonary epithelial cells compared to A549 group, and the secretion levels of IL-6 and TNF-α in the combined infection group were significantly higher than those of *P. aeruginosa* alone groups (**Figure 4C**). In addition, the single *P. aeruginosa* or *F. nucleatum* infection groups at a MOI of 200 were also used as controls. **Figure 4C** demonstrated that there was not significantly difference in the expressions of IL-1β, IL-6 and TNF-α in pulmonary epithelial cells between Pa100 and Pa200 groups, and between Fn100 and Fn200 groups.

### The Model of Pulmonary Epithelial Cells Pretreated With *P. aeruginosa*: *F. nucleatum* Subsequential Infection Maintains *P. aeruginosa*-Induced Inflammatory Cytotoxicity of Pulmonary Epithelial Cells

**Figure 5:**
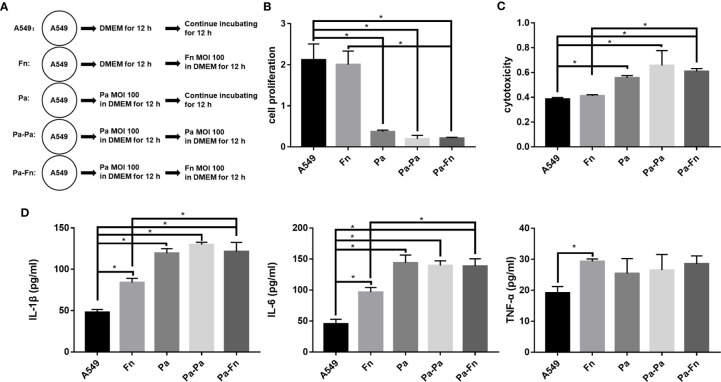
The effect of *F. nucleatum* subsequential infection on cellular proliferation, cytotoxicity and inflammation. **(A)** Schematic representation of cell assay. A549 cells cultured in DMEM for 24 h were the blank control. Fn represented A549 cells precultured in DMEM for 12 h followed by *F. nucleatum* (MOI 100) infection for other 12 h. Pa represented A549 cells treated with *P. aeruginosa* (MOI 100) for 24 h. Pa-Pa means that A549 cells pretreated with *P. aeruginosa* (MOI 100) for 12 h continue to be infected with another *P. aeruginosa* (MOI 100) for 12 h. Pa-Fn means that A549 cells pretreated with *P. aeruginosa* (MOI 100) for 12 h continue to be infected with *F. nucleatum* (MOI 100) for 12 h. **(B)** The effect of *F. nucleatum* subsequential infection on cellular proliferation of pulmonary epithelial cells. **(C)** The effect of *F. nucleatum* subsequential infection on cytotoxicity of pulmonary epithelial cells. **(D)** The effect of *F. nucleatum* subsequential infection on IL-1β, IL-6 and TNF-α productions of pulmonary epithelial cells. *P < 0.05, statistics were achieved by analysis of variance followed by Sidak’s multiple comparisons test.

To determine the role of *F. nucleatum* in the case where respiratory *P. aeruginosa* infection has already occurred, pulmonary epithelial cells were pretreated with *P. aeruginosa* followed with *F. nucleatum* infection (**Figure 5A**). **Figures 5B, C** demonstrated that single *F. nucleatum* infection did not affect the proliferation and cytotoxicity of pulmonary epithelial cell (Fn versus A549), while *P. aeruginosa* significantly inhibited the proliferation and induced cytotoxicity of pulmonary epithelial cells no matter with or without bacterial subsequential infection (Pa/Pa-Pa/Pa-Fn versus A549, **Figures 5B, C**). But, either *F. nucleatum* or *P. aeruginosa* subsequential infection just maintained the inhibition of cell proliferation and the induction of cytotoxicity caused by *P. aeruginosa* (Pa-Fn/Pa-Pa versus Pa, **Figures 5B, C**). In addition, although single *F. nucleatum* infection significantly induced the secretions of IL-1β, IL-6 and TNF-α in pulmonary epithelial cells (Fn versus A549), *P. aeruginosa* just upregulated the expressions of IL-1β and IL-6 no matter with or without bacterial subsequential infection (Pa/Pa-Pa/Pa-Fn versus A549, **Figure 5D**). The expression levels of IL-1β and IL-6 in *P. aeruginosa* and *F. nucleatum* sequential infection group were consistent with those of single *P. aeruginosa* infection group and *P. aeruginosa* subsequential infection group (Pa-Fn versus Pa, Pa-Fn versus Pa-Pa), but higher than those of single *F. nucleatum* infection group (Pa-Fn versus Fn, **Figure 5D**).

### The Model of Pulmonary Epithelial Cells Pretreated With *F. nucleatum*: *F. nucleatum* Pretreatment Enlarged TNF-α Associated Cytotoxicity of Pulmonary Epithelial Cells Subsequentially Infected With *P. aeruginosa*

**Figure 6:**
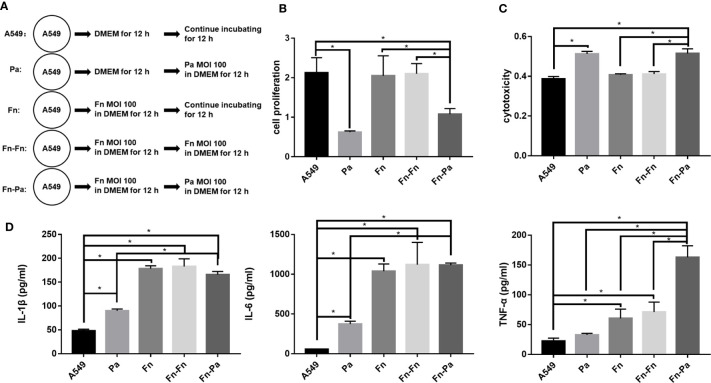
The effect of *F. nucleatum* pretreatment on cellular proliferation, cytotoxicity and inflammation. **(A)** Schematic representation of cell assay. A549 cells cultured in DMEM for 24 h were the blank control. Pa represented A549 cells precultured in DMEM for 12 h followed by *P. aeruginosa* (MOI 100) infection for other 12 h. Fn represented A549 cells treated with *F. nucleatum* (MOI 100) for 24 h. Fn-Fn means that A549 cells pretreated with *F. nucleatum* (MOI 100) for 12 h continue to be infected with another *F. nucleatum* (MOI 100) for 12 h. Fn-Pa means that A549 cells pretreated with *F. nucleatum* (MOI 100) for 12 h continue to be infected with *P. aeruginosa* (MOI 100) for 12 h. **(B)** The effect of *F. nucleatum* pretreatment on cellular proliferation of pulmonary epithelial cells. **(C)** The effect of *F. nucleatum* pretreatment on cytotoxicity of pulmonary epithelial cells. **(D)** The effect of *F. nucleatum* pretreatment on IL-1β, IL-6 and TNF-α productions of pulmonary epithelial cells. *P < 0.05, statistics were achieved by analysis of variance followed by Sidak’s multiple comparisons test.

In order to explore whether the presence of *F. nucleatum* would affect the progress of respiratory *P. aeruginosa* infection, pulmonary epithelial cells were pretreated with *F. nucleatum* followed with *P. aeruginosa* infection (**Figure 6A**). **Figures 6B, C** demonstrated that single *F. nucleatum* infection did not affect the proliferation and cytotoxicity of pulmonary epithelial cell (Fn versus A549), but *P. aeruginosa* subsequential infection significantly inhibited the proliferation and induced cytotoxicity of pulmonary epithelial cells, no matter which were pretreated with *F. nucleatum* or not (Pa versus A549, Fn-Pa versus A549/Fn). In addition, *F. nucleatum* alone significantly upregulated the secretions of IL-1β, IL-6 and TNF-α (Fn versus A549), while *P. aeruginosa* alone significantly upregulated the secretions of IL-1β and IL-6 without affecting TNF-α expression (Pa versus A549, **Figure 6D**). However, *P. aeruginosa* subsequential infection significantly enhanced the secretions of IL-1β, IL-6 and TNF-α in pulmonary epithelial cells pretreated with *F. nucleatum* compared with A549 and single *P. aeruginosa* group (Fn-Pa versus A549/Pa), but only TNF-α secretion in *F. nucleatum* and *P. aeruginosa* sequential infection group was also higher than that of *F. nucleatum* alone group (Fn-Pa versus Fn, **Figure 6D**). In order to eliminate the effect of the increment in bacterial loads on the sequential infection assay, *F. nucleatum* subsequential infection group (Fn-Fn) was included. **Figures 6B–D** demonstrated that there was not significantly difference in cell proliferation, cytotoxicity and the secretions of IL-1β, IL-6 and TNF-α in pulmonary epithelial cell between Fn group and Fn-Fn group.

## Discussion

Several studies have analyzed oral microbiome and lung microbiome of patients with respiratory infection, and demonstrate that the bacterial composition of dental plaque and lung samples is highly similar, and pathogens isolated from the lung are genetically indistinguishable from strains of the same species isolated from the oral cavity (Heo et al., 2008; Tan et al., 2014). In addition, some studies analyzing the microbiome associated with respiratory infection indicate that *F. nucleatum* widely exists in the respiratory tract of patients with respiratory diseases such as COPD, empyema and lung abscess (Nagaoka et al., 2017; Dyrhovden et al., 2019; Li et al., 2020), and *F. nucleatum* is considered as a biomarker of lung function deterioration in COPD patients coinfected with *P. aeruginosa* (Li et al., 2020). In polymicrobial diseases, there are complex interactions among different species, leading to synergisms in creating clinical symptoms and pathologies. Therefore, this study plans to explore the potential mechanism of *F. nucleatum* cooperating with *P. aeruginosa* to weaken lung function of COPD patients from two aspects: interspecies interaction between *P. aeruginosa* and *F. nucleatum*, and the interaction of *P. aeruginosa* and *F. nucleatum* with pulmonary epithelial cells.

Bacterial autoaggregation and coaggregation were defined as the adherence of bacteria belonging to the same strain and different bacterial strains, respectively, which are essential for the development of multispecies biofilm communities and the infection of host cell (Polak et al., 2012; Karched et al., 2015). This study demonstrated that *F. nucleatum*, a Gram-negative obligate anaerobe in the oral cavity, was a strong autoaggregator, which is consistent with Karched’s study (Karched et al., 2015). *F. nucleatum* expresses a variety of surface adhesins, allowing coaggregation with most oral bacteria (Kinder and Holt, 1993; Kaplan et al., 2009; Coppenhagen-Glazer et al., 2015). That may be the reason that *P. aeruginosa* coaggregates with *F. nucleatum* moderately, although *P. aeruginosa* is a poor autoaggregator. On the other hand, the adhesion and invasion of bacteria to host cells is a prerequisite for their virulence and infection. This study found that both *P. aeruginosa* and *F. nucleatum* were able to adhere to and invade into pulmonary epithelial cells, which was consistent with the previous studies (Fleiszig et al., 1994; Fleiszig et al., 1995; Han et al., 2000), and their infection number increased as the MOI of bacteria increased. Interestingly, the number of *P. aerugionosa* attaching to pulmonary epithelial cells at a MOI of 50 was similar to that at a MOI of 100, but the number of *P. aerugionosa* invading pulmonary epithelial cells at a MOI of 50 was significantly lower than that at a MOI of 100. Pan et al. have explained this phenomenon as “adhesion may not be the sole prerequisite for invasion” (Pan et al., 2009), which may also be the reason why coinfection with *P. aeruginosa* and *F. nucleatum* did not affect the adhesion of each other to pulmonary epithelial cells but enhanced each bacterial invasion. In addition, when pulmonary epithelial cells were coinfected with *P. aeruginosa* and *F. nucleatum*, the number of *P. aeruginosa* or *F. nucleatum* adhering to pulmonary epithelial cells is equal to that of the single *P. aeruginosa* or *F. nucleatum* group, respectively. That means that the total number of bacteria attached to the cell surface in the combined infection group is equivalent to the sum of the number of the single species infection groups, which is also the reason that more bacteria to coaggregate and adhere to the cell surface in the combined infection group compared with the single species groups. It has been reported that *F. nucleatum* not only enhances the adhesion and invasion of invasive bacteria such as *P. gingivalis* and *A. actinomycetemcomitans* (Saito et al., 2012; Li et al., 2015), but also transports bacteria with limited adhesion abilities such as *S. cristatus* and *S. sanguis* into the oral epithelium *via* its coaggregation function (Edwards et al., 2006). This study also demonstrated that when the MOI of bacteria was increased from 100 to 200, it did not significantly change the number of bacterial attachment and invasion. Therefore, these results suggest that coinfection with *P. aeruginosa* and *F. nucleatum* promotes each bacterial invasion into pulmonary epithelial cells due to coaggregation between two bacterial species, which may allow bacteria to evade host immune defenses, but also make host cells suffer huge damage.

After bacteria entry into host cells, some bacteria can inhibit the apoptosis of host cells to replicate and survive within the cells in order to avoid host immune defense, but certain bacteria directly induce cell death to aggravate tissue damage. Previous studies show that *P. aeruginosa* can directly act on tissue cells through its type III secretion system or toxic factors such as pyocyanin to induce apoptosis (Broquet and Asehnoune, 2015; Ranjani et al., 2015). This study demonstrated that *P. aeruginosa* attached to and destroyed the cell junction of pulmonary epithelial cells, significantly inhibited cell proliferation, and induced cytotoxic death of pulmonary epithelial cells. As distinct from *P. aeruginosa*, *F. nucleatum* promoted the proliferation of pulmonary epithelial cells without inducing cytotoxicity in the initial stage of infection (8 h). However, the presence of *F. nucleatum* transiently alleviated the inhibitory effect of *P. aeruginosa* on cell proliferation and weaken *P. aeruginosa-*induced cytotoxicity of pulmonary epithelial cells that simultaneously infected with *P. aeruginosa* and *F. nucleatum*, but simply increasing the MOI of the same bacteria could not change the effects of this bacteria on cell proliferation and cytotoxicity of pulmonary epithelial cells. Our previous study also demonstrated that periodontal pathogen *P. gingivalis* could transiently inhibit *P. aeruginosa*-induced apoptosis of pulmonary epithelial cells though the STAT3 signaling pathway (Li et al., 2014). These results indicate that *F. nucleatum* promotes *P. aeruginosa* invasion and transiently resists *P. aeruginosa*-induced damage of pulmonary epithelial cells, which may be a common characteristic of periodontal anaerobic pathogen to cause chronic persistent infection of *P. aeruginosa* in the lung of COPD patients.

It is well-known that the acute exacerbation of COPD is closely related to the persistent airway inflammation induced by bacterial infection. The expression of IL-1β in patients with COPD is upregulated, and IL-1β level is correlated with the increase of airway neutrophils and the decline of lung function (Sapey et al., 2009). Airway (sputum) IL-1β is considered to be a biomarker for acute exacerbation of COPD associated with bacteria (Bafadhel et al., 2011). Demera et al. found that the content of *P. aeruginosa* in the sputum was positively corelated with IL-1β level, and TNF-α and IL-6 are IL-1β signature and associated with IL-1β-mediated COPD exacerbation (Damera et al., 2016). This study showed that both *P. aeruginosa* and *F. nucleatum* could significantly induce IL-1β and IL-6 secretion, *F. nucleatum* also induce TNF-α secretion in pulmonary epithelial cells. In addition, the simultaneous infection with *P. aeruginosa* and *F. nucleatum* significantly enhance IL-6 and TNF-α expressions in pulmonary epithelial cells compared with single *P. aeruginosa* infection group, which were similar to respiratory syncytial virus that enhanced the release of inflammatory factors IL-6 and IL-8 in bronchial epithelial cells coinfected with *P. aeruginosa* (Bellinghausen et al., 2016). However, there was no statistical difference in the expressions of IL-1β, IL-6 and TNF-α between *F. nucleatum* group and the combined infection group, which may be due to the fact that coinfection with *P. aeruginosa* and *F. nucleatum* significantly induced cytotoxicity death of pulmonary epithelial cells at 24 h. In addition, this study also demonstrated that simply increasing the MOI of the same bacteria could not change the effects of this bacteria on the secretions of IL-1β, IL-6 and TNF-α in pulmonary epithelial cells. Fu et al. pointed out that sputum IL-1β and serum IL-6 were higher in the frequent exacerbators with COPD, which activated the IL-1β-system inflammation axis to increase the risk of frequent exacerbations of COPD patients (Fu et al., 2015). Therefore, these results suggest that the simultaneous infection with *F. nucleatum* and *P. aeruginosa* induces over-release of pro-inflammatory cytokines IL-6 and TNF-α in pulmonary epithelial cells, which may contribute to increase the susceptibility of COPD exacerbation.

It is a widely recognized clinical phenomenon that patients with more severe COPD are more likely to have severe periodontal disease (Si et al., 2012; Tan et al., 2019), and a significantly increased risk of COPD occurs among severe periodontitis subjects (Takeuchi et al., 2019). Our previous study finds that 45.3% patients with COPD exacerbation are associated with respiratory coinfection with *F. nucleatum* and *P. aeruginosa* (Li et al., 2020), two types of sequential infection model with *P. aeruginosa* and *F. nucleatum* are established to fully elaborate the role of mixed infection in COPD. The current study demonstrated that no matter with *F. nucleatum* pretreatment or subsequential infection, *P. aeruginosa* significantly induced cytotoxic damage of pulmonary epithelial cells. *F. nucleatum* pretreatment or subsequential infection kept or even amplified *P. aeruginosa*-induced cytotoxicity of pulmonary epithelial cells, which may explain why *F. nucleatum* reduced lung function of COPD patients accompanied with *P. aeruginosa* infection (Li et al., 2020). On the other hand, *F. nucleatum* subsequential infection maintained the secretion levels of IL-1β, IL-6 and TNF-α in pulmonary epithelial cells compared to the single *P. aeruginosa* group, which may be due to the low cellular viability caused by *P. aeruginosa* pretreatment. Nevertheless, *F. nucleatum* pretreatment amplified the secretions of IL-1β, IL-6 and TNF-α of pulmonary epithelial cells induced by *P. aeruginosa*, and only TNF-α secretion of *F. nucleatum* and *P. aeruginosa* sequential infection group showed a significant synergistic increase effect compared with the single species groups. In addition, sequential inoculation of the same bacteria did not significant affect the effects of this bacteria on cell proliferation, cytotoxicity and inflammation of pulmonary epithelial cells. These results suggest that *F. nucleatum* can maintain or even aggravate *P. aeruginosa*-induced inflammatory cytotoxicity of pulmonary epithelial cells in the sequential infection models, which may be the reason for *F. nucleatum* cooperating with *P. aeruginosa* to exacerbate lung injury and induce acute exacerbation of COPD.

**Figure 7:**
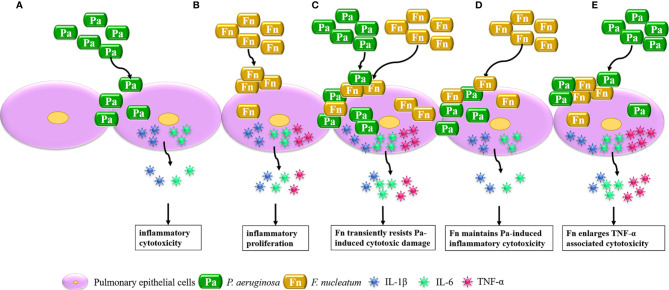
Schematic diagram of the effect of mixed infection with *P. aeruginosa* and *F. nucleatum* on pulmonary epithelial cells based on the present model. **(A)** When pulmonary epithelial cells are infected with *P. aeruginosa* alone, *P. aeruginosa* induces IL-1β and IL-6 associated inflammatory cytotoxicity of pulmonary epithelial cells. **(B)** When pulmonary epithelial cells are infected with *F*. *nucleatum* alone, *F. nucleatum* initiates high inflammatory proliferation of pulmonary epithelial cells. **(C)** When pulmonary epithelial cells are simultaneously infected with *P. aeruginosa* and *F*. *nucleatum*, *F*. *nucleatum* coaggregates with *P. aeruginosa* to synergistically invade into pulmonary epithelial cells and transiently resists *P. aeruginosa*-induced cytotoxic damage to induce over-release of IL-6 and TNF-α. **(D)** When pulmonary epithelial cells are pretreated with *P. aeruginosa*, *F. nucleatum* subsequential infection maintains *P. aeruginosa*-induced inflammatory cytotoxicity. **(E)** When pulmonary epithelial cells are pretreated with *F. nucleatum*, *F. nucleatum* pretreatment enlarged TNF-α associated cytotoxicity of pulmonary epithelial cells subsequentially infected with *P. aeruginosa*.

In conclusion, considerable research has focused on the interaction between single bacterium and host cells, while this study highlights the effect of bacterial mixed infection on cellular biological function through establishing bacterial simultaneous and sequential infection models, which is summarized in **Figure 7**. It demonstrated that *P. aeruginosa* mainly induces IL-1β and IL-6 associated inflammatory cytotoxicity of pulmonary epithelial cells, *F. nucleatum* mainly initiates high inflammatory proliferation of pulmonary epithelial cells. When pulmonary epithelial cells are simultaneously infected with *P. aeruginosa* and *F. nucleatum*, *F. nucleatum* coaggregates with *P. aeruginosa* to synergistically invade into pulmonary epithelial cells and transiently resists *P. aeruginosa*-induced cytotoxic damage, and induces over-release of IL-6 and TNF-α, which may cause a chronic persistent pulmonary infection and increase the susceptibility of COPD exacerbation. In addition, the presence of *F. nucleatum* maintains or even aggravates *P. aeruginosa*-induced inflammatory cytotoxicity of pulmonary epithelial cells in the sequential infection models, which may be the reason for *F. nucleatum* reducing lung function of COPD patients accompanied with *P. aeruginosa* infection and may contribute to persistent and/or frequent exacerbation of COPD patients accompanied with *P. aeruginosa* and *F. nucleatum* coinfection. Therefore, oral anaerobic pathogens, especially *F. nucleatum*, should never be neglected in the treatment of COPD.

## Data Availability Statement

The original contributions presented in the study are included in the article/supplementary material. Further inquiries can be directed to the corresponding author.

## Author Contributions

All authors contributed to the article and approved the submitted version. QL designed and performed the experiments, analyzed the data, wrote and revised the draft of the paper. HW, LT, SZ, LL and XT participated in the experiments, analyzed the data and revised the article. YP developed the idea for this study and revised the manuscript.

## Funding

This work was supported by the National Natural Science Foundation of China (NO. 81670997 to YP), the Plan of the talents for Liaoning Development (NO. XLYC1802129 to YP) and the Higher Education Fundamental Research Project of Liaoning Province (NO. LQNK201724 to QL).

## Conflict of Interest

The authors declare that the research was conducted in the absence of any commercial or financial relationships that could be construed as a potential conflict of interest.
